# Comparison of efficacy and safety of different fixation devices for anterior cruciate ligament reconstruction: A Bayesian network meta-analysis protocol

**DOI:** 10.1097/MD.0000000000014911

**Published:** 2019-03-22

**Authors:** Jiaxin Jin, Liping Yu, Min Wei, Yi Shang, Xin Wang

**Affiliations:** a Department of Orthopaedics, Second Hospital of Lanzhou University; b Department of Nursing, Rehabilitation Center Hospital of Gansu Province; c Department of Anesthesiology, Affiliated Hospital of Gansu University of Chinese Medicine; d Department of General Surgery, Second Hospital of Lanzhou University; e Department of Orthopaedics, First Hospital of Lanzhou University, Lanzhou, China.

**Keywords:** anterior cruciate ligament, Bayesian network meta-analysis, efficacy, fixation devices

## Abstract

**Background:**

Anterior cruciate ligament (ACL) injury is a common ligament injury to the knee joint, and often lead to limited function, osteoarthritis after knee trauma, secondary damage to meniscus and cartilage, and impaired quality of life. ACL reconstruction is the gold standard surgical treatment for ACL injury, and ligament fixation after reconstruction is the key factor of ACL reconstruction success. However, the optimal fixation device for ACL reconstruction remains unclear. This study aims to evaluate the efficacy and safety of different fixation devices and to find the best fixation device for ACL reconstruction.

**Methods:**

The PubMed, EMBASE, Cochrane Central Register of Controlled Trials (CENTRAL), and Chinese Biomedicine Literature will be searched to identify relevant studies from inception to December 2018. We will include randomized controlled trials (RCTs) comparing the effects of different fixation devices fixed on the femoral side in arthroscopically assisted ACL reconstruction. Risk of bias assessment of the included RCTs will be conducted according to the Cochrane Handbook 5.1.0. A Bayesian network meta-analysis (NMA) will be performed using R software.

**Results:**

The results of this NMA will be submitted to a peer-reviewed journal for publication.

**Conclusion:**

This NMA will summarize the direct and indirect evidence to evaluate the effect of different fixation devices for ACL reconstruction.

## 1 Introduction

The knee is composed of patellofemoral and tibiofemoral joints, the biomechanics of these joints is complicated due to the characteristics of human walking upright.^[1]^ Anterior cruciate ligament (ACL) is one of the main stable structures of the knee joint.^[2,3]^ Its main function is to limit the excessive displacement of the tibia and maintain the stability of the knee joint, thus ensuring the smooth operation of the lower limbs of the body.^[4,5]^ ACL rupture is a common ligament injury to the knee joint, usually occurring in young and physically active people.^[6–8]^ The incidence of ACL injury in Sweden is about 7000 cases per year and nearly 200,000 ACL injuries per year in the United States.^[9–11]^ With the increase in sports injuries and traffic accidents, we can predict that the incidence of ACL injuries is gradually increasing. ACL injury can lead to limited function, osteoarthritis after knee trauma, secondary damage to meniscus and cartilage, and impaired quality of life.^[12,13]^ In both male and female athletes, ACL injuries often lead to permanent disability, adding a heavy burden to their families.^[14]^

There are many treatment options for ACL injury, including nonsurgical treatments, such as physical therapy and activity modification, and surgical treatments, such as primary repair, enhanced primary repair, prosthetic replacement, and ACL reconstruction using various graft materials.^[15–18]^ ACL reconstruction with either hamstring, quadriceps, or patella tendon graft is considered to be the gold standard surgical treatment.^[19,20]^ However, ligament fixation after reconstruction of the femoral side is the key factor and weakest part of ACL reconstruction success.^[21]^ Currently, there are many fixation devices for the femoral side of the knee joint. The proximal articular surface fixation devices include screws, transverse nails such as Rigidfix, cross nails such as TransFix, Biotransfix;^[22–24]^ fixing methods away from the articular surface are portal nails and suspension fixation such as Endobutton.^[25,26]^ Recently, many traditional meta-analyses compared the efficacy, safety, adverse reactions and long-term complications of different fixation devices for graft fixation in ACL reconstruction, and some fixtures showed good effect.^[27–29]^ However, which individual fixation device or combined fixation device is the best choice for ACL reconstruction remains unclear due to lacking multiple treatment comparisons. Therefore, it is necessary to assess these fixation methods by network meta-analysis (NMA). The objectives of this Bayesian NMA are to evaluate the efficacy and safety of different fixation devices and to find the optimal fixation device for ACL reconstruction.

## 2 Methods

### 2.1 Design and registration

We registered on the international prospective register of systematic review (PROSPERO) (CRD42019119285) to publish our study protocol. We will follow the PRISMA (Preferred Reporting Items for Systematic Reviews and Meta-analysis statements) extension statement for reporting our NMA.^[30]^

### 2.2 Eligibility criteria

#### 2.2.1 Type of studies

Randomized controlled trials (RCTs) comparing the effects of different fixation devices fixed on the femoral side in arthroscopically assisted ACL reconstruction in patients will be included. At least 1 of the following parameters was reported: postoperative Lysholm score, postoperative KT-1000 arthrometry, International Knee Documentation Committee (IKDC) scores, degree of bone widening, postoperative complications. There are no language restrictions. Studies will be excluded if there are insufficient data to summarize the results after trying to contact the author about data provision and duplicate publications.

#### 2.2.2 Patients

Patients with ACL injury undergoing arthroscopic reconstruction. We will put no limitations on age, gender, and nations.

#### 2.2.3 Interventions and comparators

Any type of fixation device used for arthroscopic reconstruction of the ACL, such as Endobutton, Transfix, Biotransfix, Rigidfix, Intrafix, Aperfix, and Arthrex. There are no restrictions on the manufacturer and model of the fixation device.

#### 2.2.4 Outcome of interest

The primary outcomes of interest are the IKDC scores, Lysholm scores, and KT-1000 arthrometry. The second outcomes are adverse complications such as graft tunnel widening, degenerative change, joint effusion, synovitis, infection, graft tear, and cystic formation.

### 2.3 Information sources

A systematic search will be performed using PubMed, EMBASE, Cochrane Central Register of Controlled Trials (CENTRAL), and Chinese Biomedicine Literature to identify relevant studies from inception to December 2018. There will be no limitations on the publication languages. The reference lists of included trials and reviews identified from initial searches will be scanned for more relevant studies.

### 2.4 Search strategy

We will use search terms related to Anterior Cruciate Ligament, Fixation Device, Fasteners, Fixator, Bone Screws, Endobutton, TransFix, Biotransfix, Rigidfix, Intrafix, Aperfix, Arthrex, random, and RCT. Search strategy of PubMed was as follows:

(1) “Anterior Cruciate Ligament”[Mesh] OR “Anterior Cruciate Ligament Reconstruction”[Mesh] OR “Anterior Cruciate Ligament Injuries”[Mesh]
(2) “Anterior Cruciate Ligament”[Title/Abstract] OR “Anterior Cruciate Ligaments”[Title/Abstract] OR “Cruciate Ligament, Anterior”[Title/Abstract] OR “Cruciate Ligaments, Anterior”[Title/Abstract] OR “Ligament, Anterior Cruciate”[Title/Abstract] OR “Ligaments, Anterior Cruciate”[Title/Abstract] OR “ACL”[Title/Abstract]
(3) #1 OR #2
(4) “Surgical Fixation Devices”[Mesh]) OR “Orthopedic Fixation Devices”[Mesh]
(5) “Device, Fixation“[Title/Abstract] OR ”Devices, Fixation“[Title/Abstract] OR ”Fixation Device“[Title/Abstract] OR ”Fixation Devices“[Title/Abstract] OR ”Fasteners“[Title/Abstract] OR ”Fastener“[Title/Abstract] OR ”Fixator“[Title/Abstract] OR ”Fixators“[Title/Abstract]
(6) ”Bone Screws“[Mesh]
(7) ”Screw“[Title/Abstract] OR ”Screws“[Title/Abstract] OR ”TransFix“[Title/Abstract] OR ”Intrafix“[Title/Abstract] OR ”Aperfix“[Title/Abstract] OR Arthrex”[Title/Abstract] OR “Biotransfix”[Title/Abstract] OR “Endobutton”[Title/Abstract] OR “Rigidfix”[Title/Abstract]
(8) #4 OR #5 OR #6 OR #7
(9) “Clinical Trials, Phase II as Topic”[Mesh] OR “Clinical Trials, Phase III as Topic”[Mesh] OR “Clinical Trials, Phase IV as Topic”[Mesh] OR “Controlled Clinical Trials as Topic”[Mesh] OR “Randomized Controlled Trials as Topic”[Mesh] OR “Intention to Treat Analysis”[Mesh] OR “Pragmatic Clinical Trials as Topic”[Mesh] OR “Clinical Trials, Phase II”[Publication Type] OR “Clinical Trials, Phase III”[Publication Type] OR “Clinical Trials, Phase IV”[Publication Type] OR “Controlled Clinical Trials”[Publication Type] OR “Randomized Controlled Trials”[Publication Type] OR “Pragmatic Clinical Trials as Topic”[Publication Type] OR “Single-Blind Method”[Mesh] OR “Double-Blind Method”[Mesh]
(10) random∗[Title/Abstract] OR blind∗[Title/Abstract] OR singleblind∗[Title/Abstract] OR doubleblind∗[Title/Abstract] OR trebleblind∗[Title/Abstract] OR tripleblind∗[Title/Abstract]
(11) #9 OR #10
(12) #3 AND #8 AND #11

### 2.5 Study selection

We will import the literature search records into EndNote X8 (Yunnan University Library) literature management software. Two reviewers will independently screen and categorize all related articles, and the full-texts of any potentially eligible studies will be retrieved independently by the same authors and examined to determine whether they meet the inclusion criteria. Multiple submissions or duplicate publications will be compared, and the more detailed one will be retained. Disagreements will be resolved by consensus or by discussion with a third reviewer.

### 2.6 Data extraction

Two reviewers will independently extract the required data from the studies selected for inclusion using Microsoft Excel 2016 (Microsoft Corp, Redmond, WA, www.microsoft.com). Data will be extracted from eligible studies including author, year of publication, country of the first author, number of authors, journal name, country of journal, funding, setting, sample size, intervention characteristics, and outcomes.

### 2.7 Risk of bias assessment

Two authors will independently assess the methodological quality of the included trials using the Cochrane risk of bias assessment tool (Cochrane Handbook for Systematic Reviews of Interventions). Each item of the tool will be classified into 3 categories (low risk, high risk, and unclear risk). Any disagreement between the reviewers on the risk of bias will be resolved by discussion.

### 2.8 Statistical analysis

We will provide summaries of the intervention effects for each study by calculating risk ratios (for dichotomous outcomes) or mean differences (for continuous outcomes) and their 95% confidence interval. Fixed-effect inverse variance meta-analysis will be used for combined data when included trial results were judged sufficiently similar. Where heterogeneity could not be explained, random effects meta-analysis will be used instead. Heterogeneity across the studies will be examined using the chi-squared test and I^2^ statistic. We will consider *P* < .1 or I^2^>50% as being indicative of substantial heterogeneity.

NMAs will be conducted on both direct evidence and indirect evidence, with the benefit of randomization in each study retained and the NMA will be conducted in a Bayesian framework using a random effects model. A consistency model will be drawn for each evaluated outcome and the relative effect size of the treatment will be calculated using the mean difference for the continuous variables. The convergence will be assessed using the potential scale reduction factor and the Brooks–Gelman–Rubin method, and a value of 1 indicates a good convergence.^[31]^ The node splitting method will be used to examine the inconsistency between direct and indirect comparisons if a loop connecting 3 or more arms exist.^[32]^ If node-splitting analysis determined *P* < .05, the inconsistency model will be used for pooled analysis. Otherwise, the consistency model will be used.^[33,34]^ The analyses will be performed using R 3.5.1 (Auckland University, https://www.r-project.org/).

## 3 Discussion

To be the best of our knowledge, there are no previous studies comparing the efficacy and safety of different fixation devices for ACL reconstruction using the method of Bayesian NMA. This study will be the first NMA that summarizes direct and indirect evidence to evaluate the effect of different fixation devices. And we will also rank each fixation device according to the probability that one is superior to the other, which will help to clearly present the most appropriate fixture. We hope that the results of this NMA will help clinicians and patients make more appropriate choices when selecting fixation devices during ACL reconstruction.

## Author contributions

J.J., L.Y., and X.W. planned and designed the research. J.J., L.Y., M.W., Y.S., and X.W. tested the feasibility of the study. J.J., L.Y., and X.W. wrote the manuscript. All authors approved the final version of the manuscript.

**Conceptualization:** Jiaxin Jin, Liping Yu, Xin Wang.

**Formal analysis:** Yi Shang, Xin Wang.

**Investigation:** Jiaxin Jin, Min Wei.

**Methodology:** Jiaxin Jin, Liping Yu, Min Wei, Yi Shang, Xin Wang.

**Project administration:** Xin Wang.

**Resources:** Liping Yu.

**Software:** Yi Shang, Xin Wang.

**Supervision:** Xin Wang.

**Validation:** Min Wei, Yi Shang, Xin Wang.

**Visualization:** Jiaxin Jin, Liping Yu, Min Wei.

**Writing – original draft:** Jiaxin Jin, Liping Yu, Xin Wang.

**Writing – review & editing:** Jiaxin Jin, Liping Yu, Xin Wang.
